# Assessing the thermal stability of toolmarks casting materials through optical comparison microscopy, virtual comparison microscopy and quantitative similarity scores

**DOI:** 10.1080/20961790.2021.1991669

**Published:** 2022-03-16

**Authors:** Alaric C. W. Koh, Kai Yuan Yeoh

**Affiliations:** Forensic Chemistry and Physics Laboratory, Health Sciences Authority, Singapore

**Keywords:** Forensic sciences, casts, coronavirus, comparison microscopy, polysiloxane, thermal stability, toolmarks

## Abstract

Toolmarks, particularly those found on bulky, inaccessible or immovable items, can be recovered by casting. To allow for subsequent comparative examinations, the casting material, typically polysiloxanes or silicones, must be able to capture and preserve fine details within a toolmark accurately. To study the stability of such details after exposure to heat, toolmark casts were heated at either 60 °C for 2 h, or 90 °C for 1 h. These casts were subsequently compared to casts that had not been exposed to heat, using traditional optical comparison microscopy, as well as virtual comparison microscopy. Digitised toolmark signatures were also extracted from the casts and compared pairwise to obtain quantitative similarity scores based on cross-correlation, consecutive matching striae and Mann-Whitney U-statistic. Our results show that the fine surface details captured on all four commercial toolmark casting materials tested herein remained stable after exposure to heat. This study shows that the above heating protocols are viable viral inactivation methods for toolmark casts that are potentially contaminated with human coronaviruses, such as SARS-CoV-2. Our findings also apply to other scenarios, such as for casts that were left in a vehicle parked under the sun.

## Introduction

The Coronavirus disease 19 (COVID-19), declared as a pandemic by the World Health Organization on 11 March 2020, is caused by the severe acute respiratory syndrome coronavirus (SARS-CoV-2) [1,2]. The laboratory received a case requesting for the examination of toolmarks casts and a makeshift questioned tool, said to have been taken from a dedicated COVID-19 isolation ward. The casts and tool, which were double bagged, had not been disinfected prior to packing. The SARS-CoV-2 had been reported to remain viable on some surfaces for days [3,4], and in particular, surfaces at medical facilities housing COVID-19 patients have been found to be contaminated [5–8]. As we were requested to provide investigative findings within a few days, we thus sought methods to inactivate any virus that might be present on the submitted items, without compromising downstream comparative examinations. Methods known to inactivate human coronaviruses include the use of heat, chemical or irradiation [2,4,9–14]. We chose heat, as it would allow for inactivation without the removal of exhibits from their packaging, thereby minimising potential exposure to the virus. Commonly used protocols involved heating at 56 °C or 60 °C for a specified period of time, such as 30 min [2,9,10,13,14]. The effectiveness of heat inactivation is known to increase with temperature [4], and Pastorino et al. [14] reported that for samples with much higher viral loads, only the 92 °C–15 min protocol showed total inactivation. A mathematical model by Hessling et al. [15] suggested that though while a 60 °C–30 min protocol might suffice under “standard conditions”, for “worst-case conditions”, up to approximately 490 min at 60 °C or slightly over 20 min at 90 °C would be necessary. In view of the variability, we chose two heating protocols, viz. at 60 °C for 2 h and at 90 °C for 1 h, based on the 60 °C–30 min and 92 °C–15 min protocols [2,9,10,13,14] with an arbitrary fourfold safety factor.

While polysiloxanes are generally known to exhibit good thermal and thermo-oxidative stability [16], there was a lack of information about the effects of heat on the stability of fine surface details reproduced on toolmarks casts. In this study, we compared the toolmarks on casts that had been subjected to heat with those that had not. All casts were taken from the same toolmark, to avoid having to consider the variability between sets of toolmarks made by the same tool. The casts were first assessed visually on a traditional optical comparison microscope. As the use of 3 D virtual comparison microscopy (VCM) is an emerging area in firearm/toolmarks forensics [17], the 3 D surface topographies of the toolmarks on the casts were obtained and compared. Finally, digitised signatures of the toolmarks were extracted from the 3 D topographies, permitting objective similarity scores to be calculated.

We would like to point out that though the initial motivation for this work was for the need to implement a suitable inactivation protocol, the findings herein are also applicable to other situations in which toolmark casts could have inadvertently been exposed to higher than ambient temperatures. One example would be casts that had been left in parked vehicles under direct solar irradiation. Under such conditions, internal temperatures of up to 70 °C [18], 80 °C [19] and even 90 °C [20] have been reported, which would be within the range covered by our investigation. We do note that during ground transportation over high altitudes, packages could be exposed to temperatures as low as −15 °C [21]. It would be worthwhile to investigate the stability of casts subjected to subfreezing temperatures, though it is not within the scope of this work.

## Materials and methods

### Sample preparation

**Figure 1.**
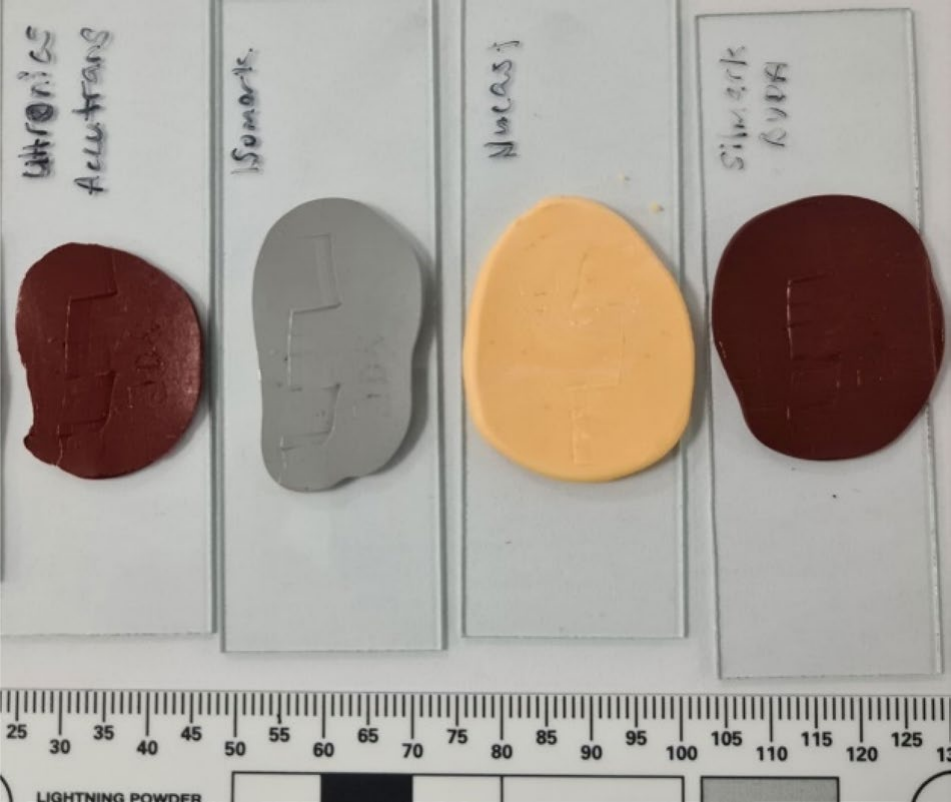
Left to right: casts made using AccuTrans® AB, Isomark™ T-1 grey, NuCASTtool, and Silmark CART.

A set of striated marks was created by dragging the tip of a screwdriver across a lead sheet. Casts of the same set of marks were made using four different products, viz. AccuTrans® AB brown silicone compound (Coltène/Whaledent AG, Altstätten, Switzerland), Isomark™ T-1 grey silicone compound (Isomark Ltd., Leicestershire, UK), NuCASTtool single use tool impression kit (Frontline Forensics Ltd., Sheffield, UK), and Silmark CART brown silicone impression compound (BVDA International BV, Haarlem, the Netherlands). An example of each is shown in Figure 1. These four products were assessed as they were available in the laboratory. The authors declare no conflict of interests, nor do we endorse any product.

Casts were heated in a Memmert UFB 500 oven (Memmert GmbH + Co. KG, Schwabach, Germany) at either 60 °C for 2 h (“60 °C–2 h” protocol) or 90 °C for 1 h (“90 °C–1 h” protocol). Samples that were not subjected to heating were used as controls for comparison.

### Optical comparison microscopy

The casts were examined under an FSC comparison microscope (Leica Microsystems CMS GmbH, Wetzlar, Germany), using a combination of LED ring light and oblique illuminations.

### Comparison of digitised toolmarks

The casts that were subjected to either heating protocol and the control casts were scanned with an InfiniteFocus XL200 G5 focus variation microscope (Alicona, Raaba/Graz, Austria) at 20× magnification, resulting in a lateral resolution of 5 μm and a vertical resolution of 0.012 μm. The regions of interest in the 3 D scans were cropped using the 3 D-Editor tool in Alicona MeasureSuite software. The cropped 3 D scans were exported to the X3P format [22] and compared using SensoCOMP software [23]. The 3 D scans were processed using the x3ptools [24] and bulletxtrctr [25] R [26] packages to compare their digitisd signatures, and to compute scores for the number of consecutive matching striae (CMS) and the cross-correlation function [27–29]. The toolmaRk [30] R package was used to compute the Mann-Whitney U-statistic of pairwise comparisons and their corresponding *P*-values, using an optimisation window of 300 and a validation window of 30 [31,32].

Both surfaces of the flat-tip of three visually similar screwdrivers, purchased from the same store, were used to create additional sets of striated toolmarks. Casts of these toolmarks were taken using the Isomark™ T-1 grey silicone compound. Three sets of digitised toolmarks were obtained for each of the six working surfaces, and processed as described above, to obtain 18 known matching scores and 135 known non-matching scores.

## Results and discussion

### Effects on comparison microscopy

**Figure 2.**
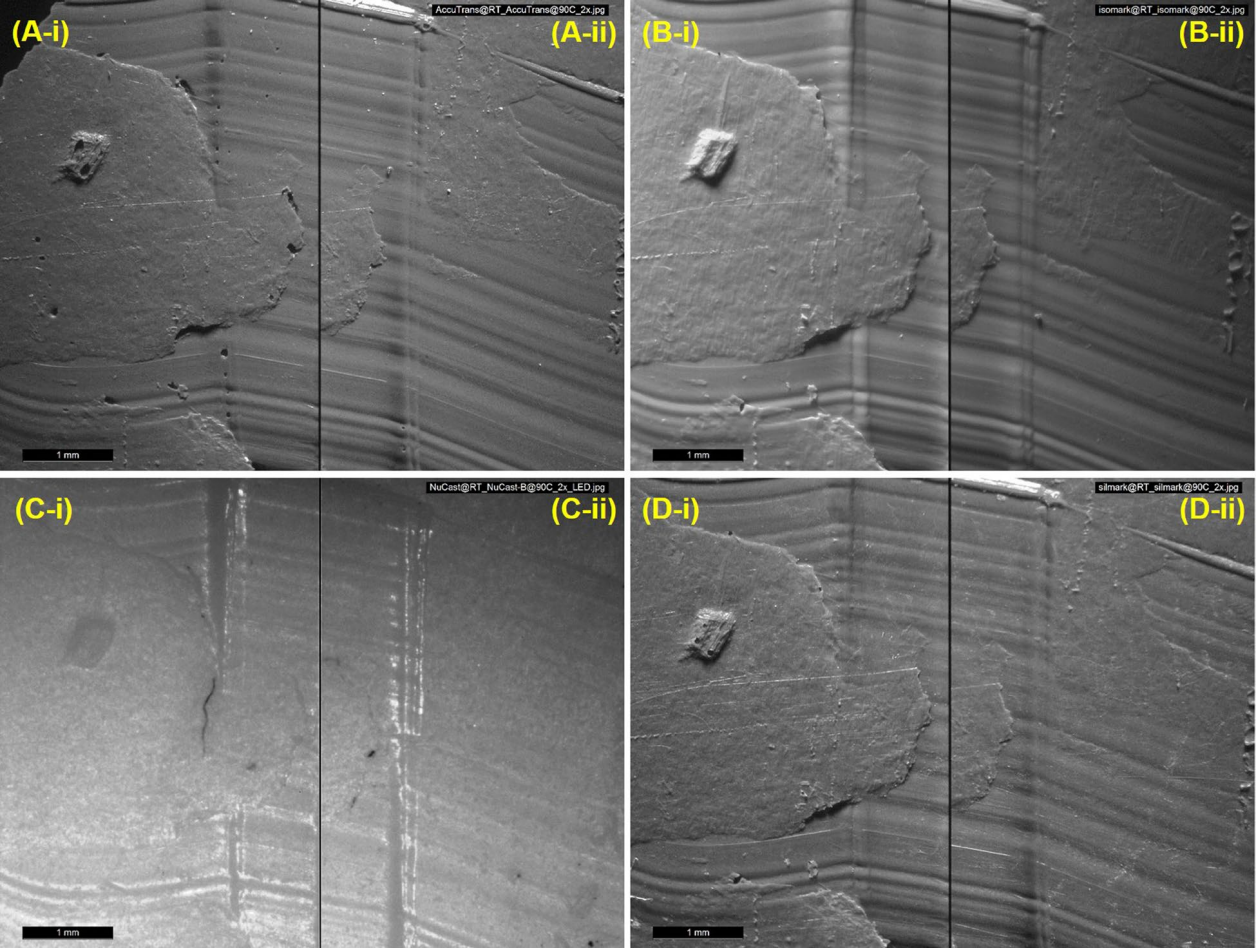
Photomicrographs showing the same set of toolmarks reproduced by (A) AccuTrans® AB, (B) Isomark™ T-1 grey, (C) NuCASTtool, and (D) Silmark CART casting materials. The casts labelled (-i) were control casts, while those labeled (-ii) were casts subjected to the 90 °C–1 h protocol. The scalebars represent 1 mm.

**Figure 3.**
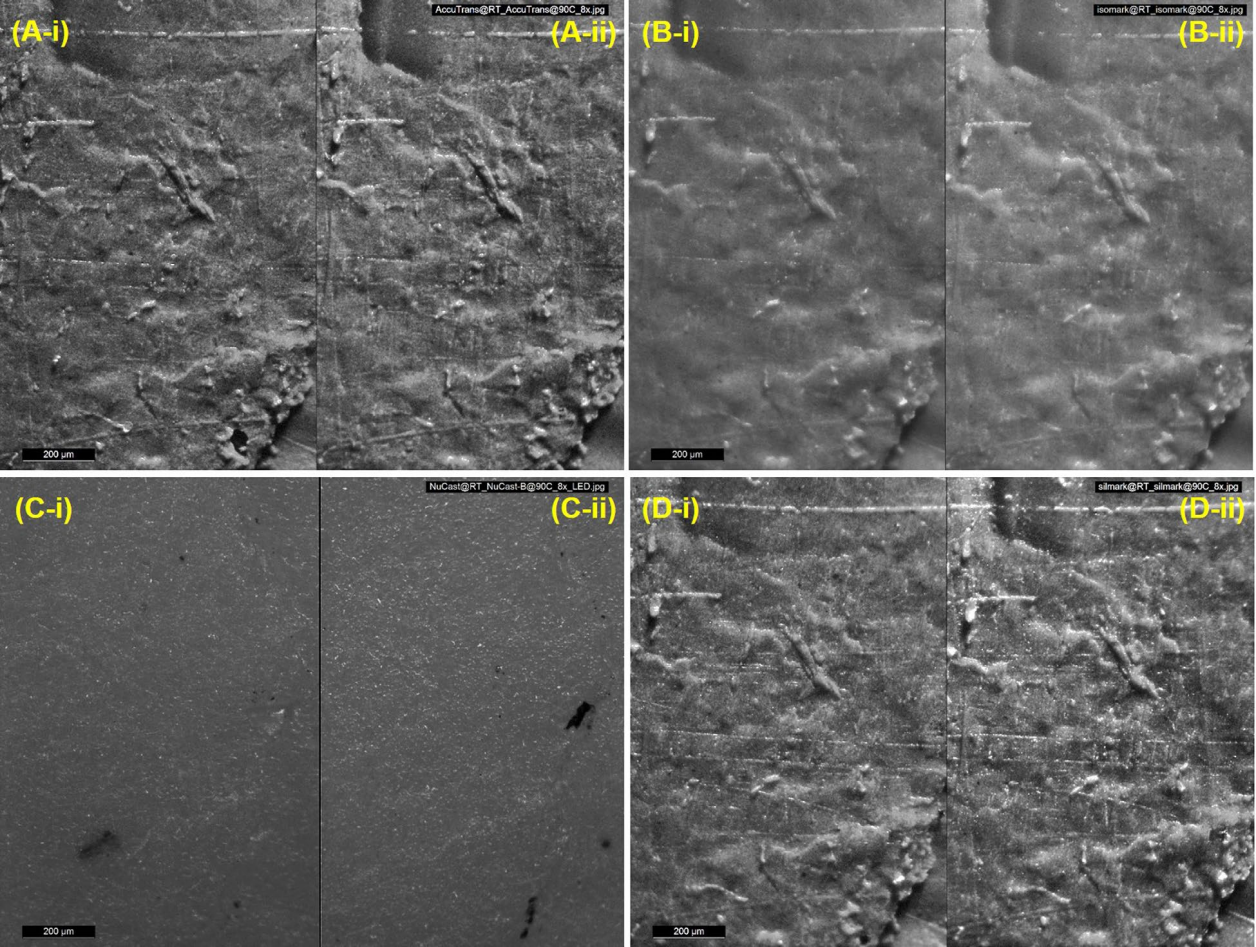
Photomicrographs showing fine surface details reproduced by (A) AccuTrans® AB, (B) Isomark™ T-1 grey, (C) NuCASTtool, and (D) Silmark CART casting materials. The casts labelled (-i) were control casts, while those labeled (-ii) were casts subjected to the 90 °C–1 h protocol. The scalebars represent 200 μm.

**Figure 4.**
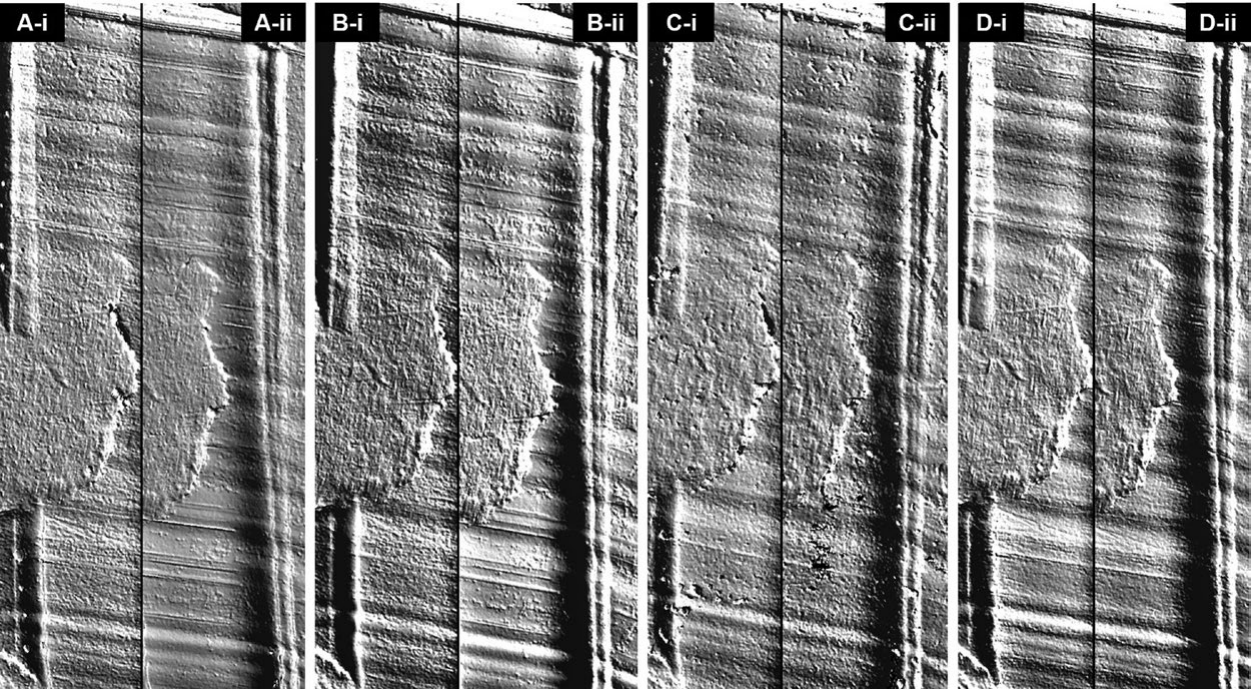
Virtual comparison microscopy of toolmarks reproduced by (A) AccuTrans® AB, (B) Isomark™ T-1 grey, (C) NuCASTtool, and (D) Silmark CART casting materials. The casts labelled (-i) were control casts, while those labeled (-ii) were casts subjected to the 90 °C–1 h protocol.

Both the heating protocols did not hinder traditional optical comparison microscopy of the toolmarks that were reproduced on the casts (Figure 2), nor led to noticeable loss or blurring of fine surface details (Figure 3). The casts were also scanned to obtain 3 D surface topographies of the same region of the recorded toolmarks. They were visualised using a VCM software and similarly, as shown in Figure 4, both heating protocols did not compromise the ability to perform comparisons.

While it was not the initial focus of this paper, NuCASTtool appeared visually to fare the poorest in terms of reproduction of marks when traditional optical comparison microscopy was employed (Figures 2 and 3). With VCM though (Figure 4), the visualisations of the marks on all four products were of comparable quality. This suggests that the visually poorer performance of NuCASTtool with traditional optical comparison microscopy was not the result of a poor reproduction of fine details, but instead, due to its lighter colour (Figure 1), which gave a poorer contrast. This demonstrates the advantages of VCM, where the scans are rendered with respect to its topography rather than its actual surface colour, as well as controlled directional virtual lighting that may be applied for rendering. We did note that to obtain the 3 D scans, the lighter surface of NuCASTtool required the use of shorter exposure times (145 μs, compared to 167 μs for Isomark™ and 425 μs for the other two).

The observed stability of the details captured on the casts with heat was not unexpected, given the reported thermal and thermo-oxidative stability of polysiloxanes [16]. We note that our observations are consistent with reports by Corso et al. [33], Pant et al. [34] and others [35,36] on the dimensional stability of dental impression materials. For example, Corso et al. [33] studied the effect of storage temperatures ranging from 4 °C to 40 °C on the dimensional stability of a polyvinyl siloxane dental impression material and found that the “*overall dimensional changes were extremely small*”.

### Similarity scores

**Figure 5.**
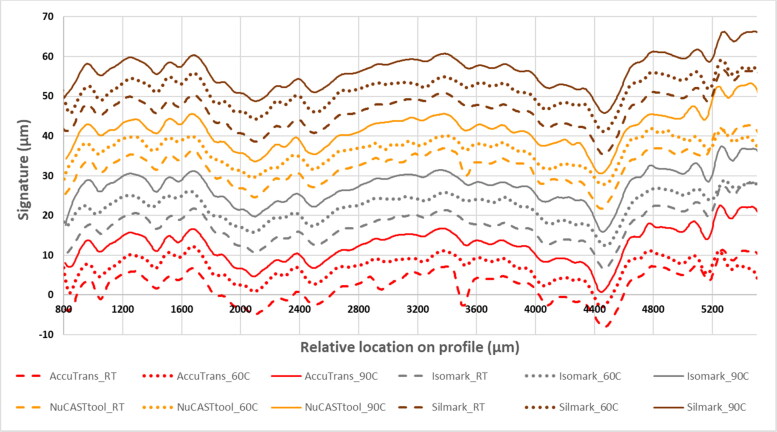
Digitised signatures of the toolmarks reproduced on AccuTrans® AB (red profiles), Isomark™ T-1 grey (grey profiles), NuCASTtool (yellow profiles), and Silmark CART (brown profiles) casts. Dashed lines were profiles of the marks on control casts, while the dotted and solid lines represent profiles of the marks on casts subjected to the 60 °C–2 h and 90 °C–1 h protocols, respectively.

The 3 D scans were processed to extract 2 D digitised signatures of the toolmarks. As shown in Figure 5, the digitised signatures of the four control casts and the eight casts subjected to heat were visually similar, suggesting that the details captured by the casts were relatively stable with heat. These digitised signature profiles were then compared pairwise, and similarity scores were computed.

**Figure 6.**
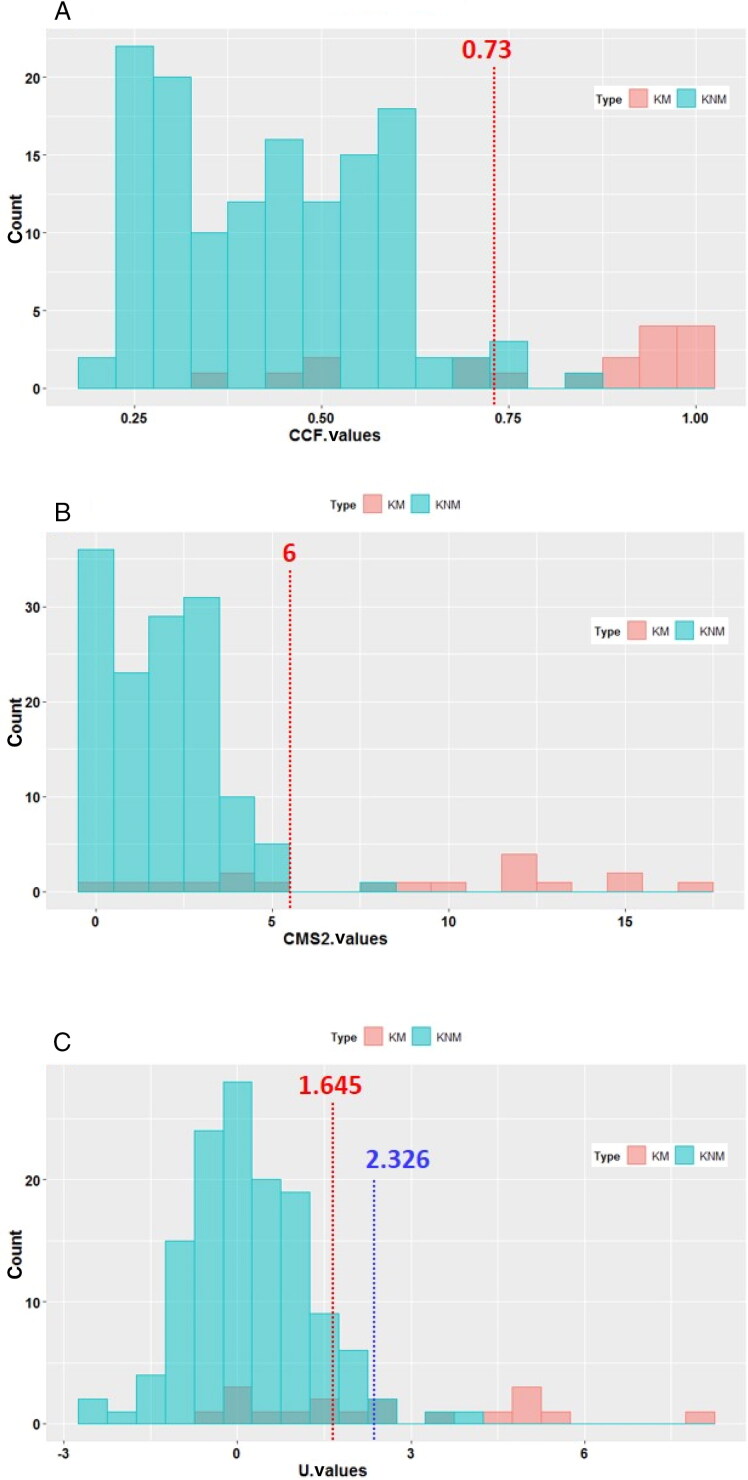
Distribution of similarity scores obtained for 18 known matching and 135 known non-matching marks from three screwdrivers: (A) CCF values, with the cut-off value of 0.73 (Table 1) indicated by the red line. (B) CMS scores, with the cut-off value of 6 (Table 1) indicated by the red line. (C) *U*-statistic scores, with the cut-off values of 1.645 and 2.326 (Table 2) indicated by the red line and the blue line, respectively. KM, known matching; KNM, known non-matching. CCF: cross-correlation function; CMS: consecutive matching striae.

Two such similarity scores, namely the cross-correlation function (CCF) and the maximum consecutive matching striae (CMS), are shown in Table 1. The CCF value can be used to assess the degree of similarity between two profiles, or to assess differences resulting from modifications to the same profile [37]. This function was applied by Zheng et al. [38] to compare toolmarks produced from consecutively manufactured chisels. Therein, the authors established a critical CCF value of 0.564, i.e. two given profiles were deemed to be a “match” if their computed correlation was greater than 0.564. The computed CCF values in this work ranged from 0.59 to 0.99, which are all greater than 0.564. The computed same-brand CCF scores ranged from 0.59 to 0.99, which was slightly wider than the range of 0.62 to 0.99 obtained for different-brand CCF scores. However, based on the known matching and known non-matching distributions that we obtained with toolmarks from the three screwdrivers (Figure 6A), the 0.564 value would not be as appropriate. Instead, a reference cut-off score of 0.73 was applied, corresponding to the 99^th^ percentile known non-matching score. There were eight scores that fell below this cut-off, all of which involved a comparison with the NuCASTtool cast subjected to the 60 °C–2 h heating protocol. Overall, the majority (38 of 66) of the values are above 0.90, where 1 is the CCF value for identical profiles.

**Table 1.** Pairwise comparison of the digitised signatures.

	AccuTrans-RT	AccuTrans-60C	AccuTrans-90C	Isomark-RT	Isomark-60C	Isomark-90C	NuCAST-RT	NuCAST-60C	NuCAST-90C	Silmark-RT	Silmark-60C	Silmark-90C
AccuTrans-RT		0.80	0.98	0.96	0.89	0.98	0.96	0.64	0.92	0.97	0.95	0.97
AccuTrans-60C	14		0.80	0.76	0.79	0.84	0.77	0.88	0.74	0.77	0.89	0.77
AccuTrans-90C	15	16		0.99	0.89	0.99	0.94	0.64	0.96	0.99	0.94	0.99
Isomark-RT	11	13	13		0.86	0.96	0.95	0.62	0.95	0.98	0.91	0.99
Isomark-60C	12	10	10	8		0.89	0.85	0.92	0.81	0.88	0.98	0.88
Isomark-90C	13	13	13	13	12		0.92	0.64	0.93	0.96	0.95	0.97
NuCAST-RT	13	15	15	11	8	8		0.62	0.92	0.94	0.91	0.94
NuCAST-60C	5	11	5	3	2	6	7		0.59	0.64	0.93	0.63
NuCAST-90C	11	13	13	9	12	12	12	7		0.96	0.86	0.97
Silmark-RT	15	15	15	12	13	13	10	10	10		0.93	0.99
Silmark-60C	13	13	13	10	11	11	12	4	10	12		0.92
Silmark-90C	14	15	15	11	13	13	12	10	10	15	10	

*Note:* Control casts are suffixed with “RT”, while those subjected to the 60 °C–2 h and 90 °C–1 h protocols are suffixed with “60C” and “90C", respectively. CCF and CMS scores are presented in blue and green, respectively. Pink cells represent comparisons which fall below a CCF score of 0.73 or CMS score of 6. CCF, cross-correlation function; CMS, consecutive matching striae.

The other similarity score, CMS, presented in Table 1 ranged from 2 to 16. The computed same-brand CMS scores ranged from 7 to 16, which was slightly better than the range of 2 to 15 obtained for different-brand scores. A majority of the scores (61 of 66) are sufficient to satisfy the conservative quantitative criteria of a single group of six or more CMS proposed by Biasotti and Murdock [39], as applied to the identification of 3D toolmarks. In our study with the screwdrivers, this criterion was also deemed to be appropriate (Figure 6B). Again, all the scores that fell under the criterion were from the NuCASTtool cast subjected to the 60 °C–2 h heating protocol, when it was compared to casts made by the other products. Practically, when examined through a traditional optical comparison microscopic, the application of this CMS criterion is achieved by counting the number of peaks on two sets of toolmarks that are aligned. Chu et al. [40] applied this concept to the comparison of peaks of digitised bullet signatures, and found that all 12 960 known non-match comparisons performed had four or fewer CMS, while 93 of 180 known match comparisons met the CMS criteria. It should be pointed out that as the “cms” score in the bulletxtrctr package [27] considers both peaks and valleys of the digitised signatures, it is not directly comparable to the criteria proposed by Biasotti and Murdock [39], nor to the results reported by Chu et al. [40]. As such, the CMS scores presented herein were instead scores for “cms2” features, defined by the authors of the bulletxtrctr package as the “number of consecutively matching elevated striation marks".

Both sets of quantitative similarity scores obtained above provide support for the visual observations that the heating protocols did not lead to the loss of surface details that would impact the ability to perform comparison microscopy. The digitised signatures were further compared using a statistically based algorithm first described by Chumbley et al. [31] and improved upon by Hadler and Morris [32]. Hadler and Morris demonstrated the applicability of a Mann-Whitney *U*-statistic based algorithm by comparing the toolmarks produced by 50 sequentially manufactured screwdriver tips, using critical values of 1.645 and 2.326, which corresponded to false-positive error rates of 0.05 and 0.01. This means that a computed *U*-statistic value greater than 1.645 (or 2.326), with an associated *P*-value smaller than 0.05 (or 0.01), would indicate that the null hypothesis of a non-match is rejected, if a false-positive error rate of 0.05 (or 0.01) is acceptable. Based on the distributions obtained with the screwdrivers, these values were deemed to be suitable cut-offs (Figure 6C). The results of our comparisons are presented in Table 2. The comparisons that fall within the critical *U*-statistic value of 1.645, i.e. at an acceptable false-positive error rate of 0.05, were highlighted in pink. All the five comparisons highlighted in pink involved the NuCASTtool cast subjected to the 90 °C–1 h heating protocol, when it was compared to casts made by the other products. Taken together with the CMS similarity scores, this casting material could be more susceptible to effects of heat than the other products. Notwithstanding, both NuCASTtool casts that were subjected to heat still performed satisfactorily when assessed using two of the three similarity scores.

**Table 2.** Pairwise comparison of the digitised signatures.

	AccuTrans-RT	AccuTrans-60C	AccuTrans-90C	Isomark-RT	Isomark-60C	Isomark-90C	NuCAST-RT	NuCAST-60C	NuCAST-90C	Silmark-RT	Silmark-60C	Silmark-90C
AccuTrans-RT		1.2E-05	4.5E-02	4.5E-05	4.9E-07	1.5E-04	5.3E-04	1.2E-05	1.4E-01	9.9E-04	5.9E-05	2.9E-04
AccuTrans-60C	4.23		1.4E-05	5.4E-06	1.1E-02	3.0E-04	4.7E-04	2.2E-06	2.2E-02	2.0E-02	2.8E-05	1.3E-02
AccuTrans-90C	1.69	4.20		2.5E-04	1.6E-02	1.4E-02	8.5E-07	1.9E-02	2.9E-01	3.9E-10	6.3E-06	3.5E-03
Isomark-RT	3.92	4.40	3.48		1.9E-04	1.7E-05	9.8E-07	1.7E-03	3.0E-05	3.0E-06	4.6E-05	2.7E-03
Isomark-60C	4.90	2.28	2.15	3.56		3.9E-02	1.8E-02	2.5E-03	1.0E-03	7.3E-03	2.3E-03	1.8E-03
Isomark-90C	3.62	3.43	2.20	4.14	1.76		3.6E-05	2.0E-03	3.8E-01	6.2E-04	1.7E-04	4.4E-04
NuCAST-RT	3.27	3.31	4.79	4.76	2.10	3.97		5.4E-03	4.5E-03	6.3E-06	1.7E-03	6.1E-04
NuCAST-60C	4.23	4.59	2.07	2.92	2.80	2.88	2.55		4.0E-03	2.8E-04	1.9E-04	3.2E-04
NuCAST-90C	1.06	2.02	0.56	4.01	3.09	0.30	2.61	2.65		1.6E-01	3.0E-02	9.0E-02
Silmark-RT	3.09	2.06	6.15	4.53	2.44	3.23	4.36	3.45	1.02		2.3E-05	6.9E-06
Silmark-60C	3.85	4.03	4.37	3.91	2.84	3.59	2.92	3.56	1.88	4.07		4.9E-06
Silmark-90C	3.44	2.22	2.70	2.78	2.92	3.33	3.24	3.41	1.34	4.35	4.42	

*Note:* Control casts are suffixed with “RT", while those subjected to the 60 °C–2 h and 90 °C–1 h protocols are suffixed with “60C” and “90C", respectively. The Mann-Whitney *U*-statistic and its associated P-value are presented in green and blue, respectively. Pink cells represent comparisons which fall within the critical U-statistic value of 1.645, while orange cells represent those with values between 1.645 and 2.326.

If the acceptable false-positive error rate is lowered to 0.01, there were 11 other comparisons, highlighted in orange, that fell within the critical *U*-statistic value of 2.326. This suggests there could be some effects of heat on these casts, which were not visually apparent with comparison microscopy, nor through the computation of CCF and CMS similarity scores, though a majority of the comparisons (ca. 92% and 76% at false positive error rates of 0.05 and 0.01, respectively) would still be considered as matching toolmarks. We did note that the *U*-statistic algorithm appeared to be sensitive to lengths of windows used for the optimisation and validation steps. Hadler and Morris [32] had mentioned that the window used for the optimisation step is generally on the order of 10% of the digitised toolmark lengths. While a recommended length of the window used for the validation step was not stated, it was given a default value corresponding to 10% of the optimisation window length in the code. When an optimisation window of ca. 10% of the digitised toolmark lengths was used in this study, a few “NA” results were obtained, e.g. for the AccuTrans-RT/AccuTrans-90C and the NuCast-RT/Isomark-90C pairs. As such, we adopted an optimisation window of length 300 instead, which was ca. 20% that of the digitised toolmarks. This resulted in a decrease in the scores for some pairs (e.g. the *U*-statistic value for Isomark-90C/NuCAST-90C decreased from 4.75 to 0.30), and an increase for some others (e.g. that of AccuTrans-90C/Silmark-RT increased from 0.89 to 6.15). For this work, the effects of further varying the lengths of the two windows were not further optimized nor investigated.

## Conclusions

Toolmarks casting materials were heated in an oven at either 60 °C for 2 h, or 90 °C for 1 h, to investigate the effects of heat on downstream examinations. The heating protocols did not lead to noticeable loss of fine details for all four types of casting materials used, and the ability to perform either traditional optical comparison microscopy or virtual comparison microscopy was not affected. These observations were supported by quantitative similarity scores computed based on the CCF and the CMS criteria, and to a certain extent, an algorithm based on the Mann-Whitney *U*-statistic. Our study suggests that toolmark casts are relatively stable when exposed to temperatures of up to 90 °C, for instance, if they were to be kept in a parked vehicle under direct sunlight. In addition, the heating protocols investigated in this study could be considered viable means to inactivate human coronaviruses, such as SARS-CoV-2, on contaminated casts.
